# Hybrid QM/MM Simulations Confirm Zn(II) Coordination Sphere That Includes Four Cysteines from the P2 × 4R Head Domain

**DOI:** 10.3390/ijms22147288

**Published:** 2021-07-07

**Authors:** Francisco Andrés Peralta, J. Pablo Huidobro-Toro, Raúl Mera-Adasme

**Affiliations:** 1 Institute for Advanced Studies, University of Strasbourg (USIAS), 67083 Strasbourg, France; fperalta@unistra.fr; 2 Departamento de Biología, Facultad de Química y Biología, Universidad de Santiago de Chile (USACH), Santiago 9170124, Chile; 3 Centro Para el Desarrollo de Nanociencia y Nanotecnología, (CEDENNA), Universidad de Santiago de Chile (USACH), Santiago 9170124, Chile; 4 Departamento de Ciencias del Ambiente, Facultad de Química y Biología, Universidad de Santiago de Chile (USACH), Santiago 9170124, Chile

**Keywords:** P2X4R head domain, QM/MM simulations, Cys as Zn(II) ligands, P2X4R Zn(II) binding site, P2X4R head domain Cys mutants

## Abstract

To ascertain the role of Zn(II) as an allosteric modulator on P2X4R, QM/MM molecular dynamic simulations were performed on the WT and two P2X4R mutants suggested by previous electrophysiological data to affect Zn(II) binding. The Gibbs free energy for the reduction of the putative P2X4R Zn(II) binding site by glutathione was estimated at −22 kcal/mol. Simulations of the WT P2X4R head domain revealed a flexible coordination sphere dominated by an octahedral geometry encompassing C126, N127, C132, C149, C159 and a water molecule. The C132A mutation disrupted the metal binding site, leading to a coordination sphere with a majority of water ligands, and a displacement of the metal ion towards the solvent. The C132A/C159A mutant exhibited a tendency towards WT-like stability by incorporating the R148 backbone to the coordination sphere. Thus, the computational findings agree with previous experimental data showing Zn(II) modulation for the WT and C132A/C159A variants, but not for the C132A mutant. The results provide molecular insights into the nature of the Zn(II) modulation in P2X4R, and the effect of the C132A and C132A/C159A mutations, accounting for an elusive modulation mechanism possibly occurring in other extracellular or membrane protein.

## 1. Introduction

Zn(II) was a relatively abundant divalent metal in prebiotic environments [1], particularly in the oceans, where life is claimed to have originated. In the course of evolution, Zn (II) was chosen over other divalent trace metals as an enzyme catalyst, a protein allosteric modulator including receptor channels, and became a structural protein determinant (as with Zn(II) fingers) with a multiplicity of cellular functions. Therefore, we deem that Zn(II) was likely an early participant in the history of life. As to why Zn(II) was selected over other abundant divalent metals, known as trace metals, we can speculate that based on its reduced redox potential it was less chemically damaging than Cu(II), for example, while acknowledging that both Zn(II) and Cu(II) accomplish relatively similar roles as protein modulators [2]. It has been recognized for more than 30 years that Zn(II) in the brain is stored in synaptic vesicles [3,4] together with transmitter molecules such as glutamate or ATP, and the metal is released to the synaptic space [5] together with transmitters where the role of this metal is still uncertain. We deem that among other properties, Zn(II) participates in brain signaling either as an agonist of ionic channels, (the Zn(II)-activated channels), or as a synapse channel modulator [6,7,8].

Zinc dyshomeostasis is thought to be involved in the pathogenesis of several diseases affecting the nervous system [9]. Our group and others have extensively examined the role of trace metals such as Zn(II) or Cu(II) as allosteric modulators of ATP-gated P2X receptor channels [10,11,12,13,14,15,16,17,18,19] and of enzymes such as copper, zinc superoxide dismutase (SOD1).

In the case of P2XRs, we have shown experimentally that different trace metals result in differences in the final modulator response [10,20,21]. While Zn(II) in the P2X4R is a positive allosteric modulator characterized by leftwards ATP concentration curves displacements, Cu(II) is a negative P2X4R allosteric modulator acting as a noncompetitive antagonist. Our findings allowed us to infer that the Zn(II) binding site must be different from the Cu(II) site and, more importantly, the final conformational change induced by these trace metals in the P2X4R TD model must be substantially distinct and differentiable. Although in the P2X2R variant, Zn(II) is known to act as a positive allosteric modulator, the determination of the Zn(II) binding site in the P2X4R, and the structural mechanism of the modulation remain unknown. We are convinced that understanding the mode of Zn(II) action will help elucidate its role in the system and might provide useful insight to other Zn(II)-dependent biological processes.

To gain understanding into the site and mechanism of Zn(II) positive allosteric modulation, recently Peralta and Huidobro-Toro [20] proposed that cysteine residues in the head domain of the P2X4R are key players in the Zn(II) mode of action as an allosteric modulator. In this model, a two-step process was elucidated. Zn(II) might bind to the immediate vicinity of the P2X4R head domain where four cysteines are found likely forming two disulfide bridges (SS2: C126-C149; SS3: C132-C159). Next, the metal becomes close to these cysteines, which might be oxidized or not, and forms a coordination complex with a pair of these cysteines. This model involves a cysteine from each disulfide bond, which as a final result elicits the P2X4R conformational changes compatible with the positive trace metal allosteric modulator role.

In this research project, we employed computational chemistry tools, at the quantum-mechanics and hybrid quantum-mechanics/molecular mechanics (QM/MM) levels of theory, to elucidate the structure of the Zn(II) coordination complex with the head domain cysteines in an attempt to further understand the molecular mechanism of Zn(II) P2X4R allosteric modulation. In addition to performing simulations on the wild type P2X4R, we also examined two P2X4R mutants, the C132A, which abolishes the allosteric modulation, and the double C132A/C159A mutant, which was reported to conserve the Zn(II) modulation [20]. Our results contribute structural information consistent with the electrophysiological report by Peralta and Huidobro-Toro (2020). In this report, we provide insight concerning the metal coordination sphere in the P2X4R head domain identifying the putative protein ligands. and an explanation to account for the sensitivity of the double Cys mutant to Zn(II) allosteric modulation despite the fact that key Cys residues were artificially mutated. The experience gained in this project is applicable to other proteins where Zn(II) plays a modulator role, helping clarify why this metal plays such a necessary role in sustaining life.

## 2. Results

### 2.1. Redox Potential of the Putative Zn(II) Binding-Site of P2X4R

Although the P2X4R protein in solution, including its head domain structure, is in an oxidized state, NEM alkylation experiments suggest that cysteines C126, C132, C149 and C159, comprising the Zn(II) site, could physiologically be reduced [20]. In order to investigate this possibility, we estimated the redox potential of the four cysteine thiols reduction by glutathione. Glutathione was chosen not only for its physiological antioxidant relevance, but also because it contains a reactive thiol group similar to the key cysteine residues in our study, which mitigate the systematic error in the method used. The Gibbs free energy for the reduction reaction of the four cysteines was obtained at the GFN2-xTB level of theory with a dielectric model for the solvent and vibrational corrections, as detailed in the Materials and Methods section, and was found to be favorable at −22 kcal/mol, equivalent to a 0.24 V reduction potential. We concluded that a reduced site is a relevant form in equilibrium, and it is thus likely to be available for Zn(II) binding.

### 2.2. QM/MM Molecular Dynamics Simulations of the WT and Mutant Head Domain of the P2X4R Protein

Several reports indicate that while the wild type P2X4R is modulated by Zn(II), the C132A mutant is resistant to the Zn(II) modulation. Curiously, it was recently reported that the double mutant C132A/C159A rescues the WT phenotype, and is modulated by Zn(II), a behavior difficult to reconcile with the hypothesis that the Zn(II) ion binds to these cysteines in the head domain producing the receptor’s response to the metal. To elucidate the Zn(II)-binding properties of the putative Zn(II) site in the head domain of P2X4R, we performed three 30 ps hybrid quantum/classical (QM/MM) molecular dynamics simulations, as detailed in the Materials and Methods section.

**Figure 1:**
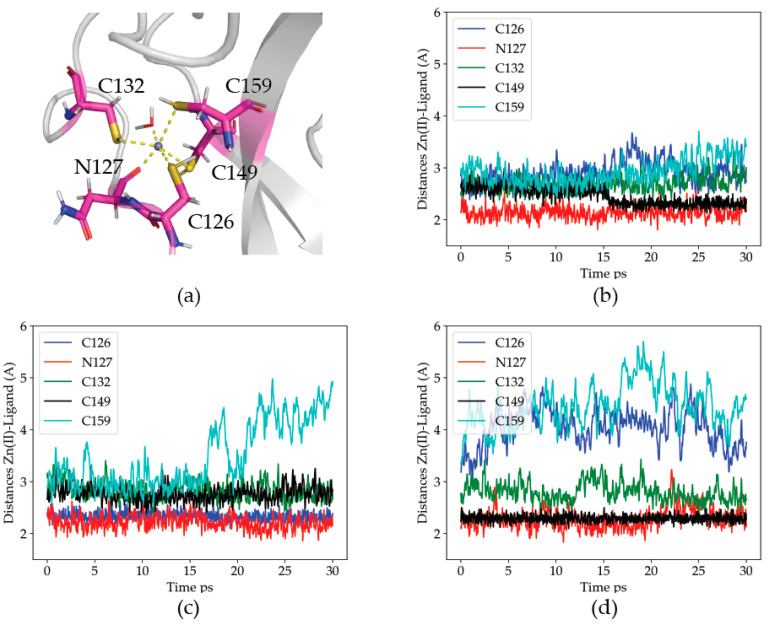
Zn(II) coordination in the optimized WT structure for the head domain of P2X4R. (**a**) Identified Zn(II) coordination ligands. (**b**–**d**) Distances from the ligands to the Zn(II) ion along each of the three 30 ps QM/MM MD trajectories for the WT head domain of P2X4R. Blue: C126, Red: N127, Green: C132, Black: C149 and Cian: C159.

Visual inspection of the trajectories shows that Zn(II) binding to the proposed site is stable upon geometry optimization and within the MD simulations, although the simulations are short, due to their very high computational cost of quantum-mechanical calculations when compared to purely classical methods (Figure 1a). The coordination is approximately octahedral, and comprises four cysteine’s thiols acting as ligands, plus the backbone-carbonyl group of the residue N127 and a water molecule.

#### 2.2.1. Dynamics of the Zn(II) Site in the Wild Type P2X4R

In order to analyze the dynamics of the coordination site, the distances from the Zn(II) ion to its protein ligands were measured. The results are given in Figure 1b–d.

Distance analysis shows that Zn(II) maintains at all times at least three protein ligands. The details of the coordination site are, nevertheless, dynamic, suggesting a relatively labile site. In the three simulations, one of the cysteine residues (either C126 or C149) becomes closer to the metal than the others. The reason for the difference is a deprotonation of the respective thiol group, where a proton is transferred to water, and the cysteine residue becomes a charged ligand. The tendency of C126, and particularly, C149, to lose the thiol proton is probably due to their proximity to the solvent bulk (each on an opposite side of the metal site). The C132 residue, although never protonated, is stable in the coordination sphere, as is the N127 residue. The least stable ligand is C159, which abandons the coordination site in two out of the three simulations. We propose that the reduced stability of C159 (upper right corner of Figure 1a) is related to its diametrically opposed position to the closer N127 ligand, which pulls the Zn(II) away from it. Altogether, the results support the critical role of these four head domain thiols in the binding and coordination of Zn(II) at this metal site.

#### 2.2.2. Dynamics of the C132A Mutant Zn(II) Site

**Figure 2:**
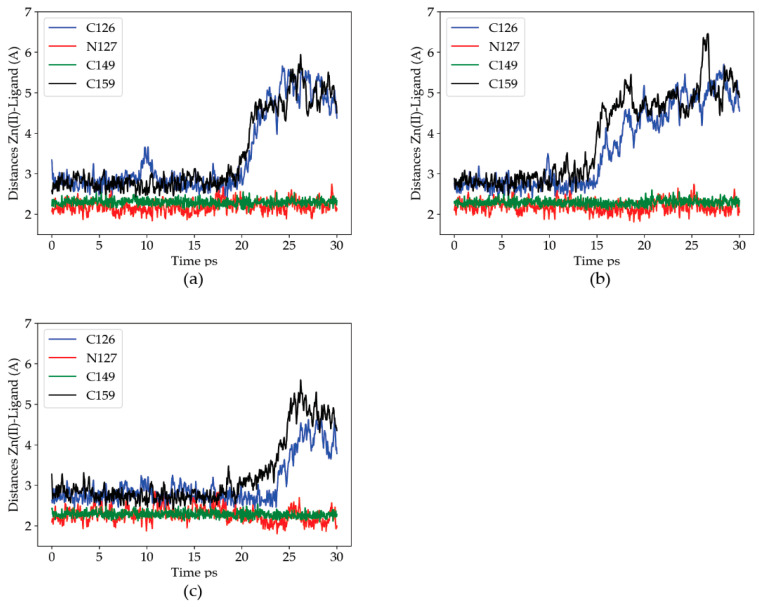
Distances for the identified Zn(II) ligands to the metal ion along each of the three 30 ps QM/MM MD trajectories for the C132A mutant of the P2X4R head domain. (**a**–**c**) Zn(II)-Ligand distances for three different trajectories. Blue: C126, Red: N127, Green: C132, Black: C159.

**Figure 3:**
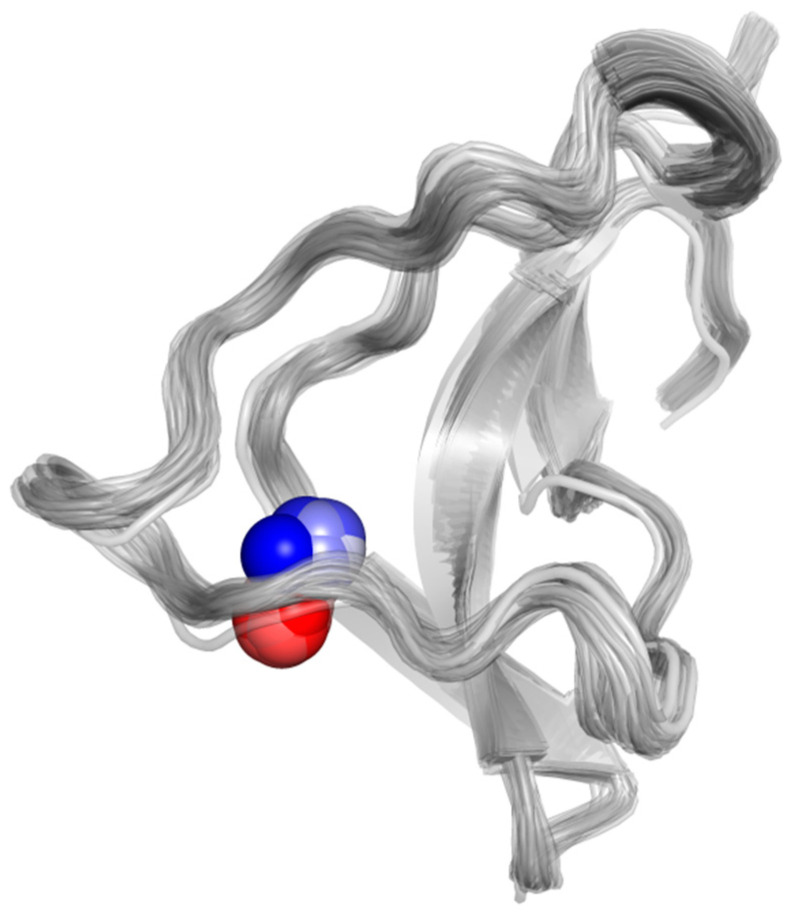
Displacement of the Zn(II) ion towards the solvent bulk along one of the three 30 ps C132A QM/MM MD trajectories. The different positions of the Zn(II) ion are shown with spheres colored from blue (for the position at the beginning of the simulation) to white, to red (at the end of the simulation). Equivalent figures for the other mutant trajectories are given in the Figure S1.

To analyze the effect of Zn(II) binding to the C132A mutation, the distances from the metal to the Zn(II) ligands found in the previous section (C126, N127, C149 and C159), along MD trajectories for the mutant structure were measured. Figure 2 shows the Zn(II)-ligand distances. In this mutant, the trajectories were fairly consistent regarding the Zn(II) site. As in the WT case, C149 is deprotonated and, together with N127, is the closest and only stable metal ion ligand. During the three simulations, both C126 and C159 abandon the metal binding site. In two out of the three trajectories, C159 is the first to detach from the coordination sphere, in agreement with the high lability exhibited by the residue in the wild type trajectories. As the two critical thiols leave the metal coordination sphere, the cation is displaced towards C149, i.e., towards the solvent bulk (see Figure 3). The data indicates a partial dissociation of the Zn(II) ion from its coordination site, in agreement with the experimental finding that the C132A mutant is no longer modulated by Zn(II).

#### 2.2.3. Dynamics of the C132A/C159A Mutant of the Zn(II) Site

**Figure 4:**
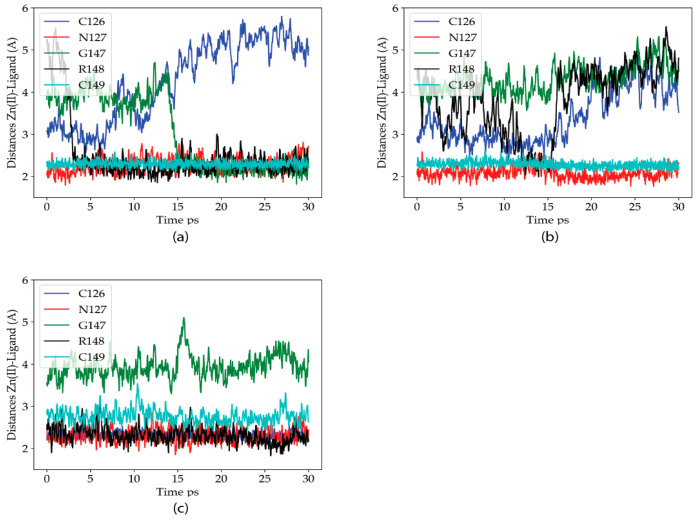
Zn(II) coordination distances for the identified Zn(II) ligands in the P2X4R head domain, along each of the three 30 ps QM/MM MD trajectories for the C132A/C159A mutant variants of the protein. (**a**–**c**) Zn(II)-Ligand distances for three different trajectories. Blue: C126, Red: N127, Green: G147, Black: R145 and Cian: C149.

QM/MM MD simulations were next performed in the P2X4R double mutant to analyze how and why this mutation restores the metal sensitivity as reported by Peralta y Huidobro-Toro (2020). Figure 4 shows the Zn(II)-ligand distances for the Zn(II) ligands along the three trajectories of this mutant. The behavior of the Zn(II) site is the most variable of the three variants studied. Nevertheless, in the three trajectories it is clearly observed that the R148 residue enters the coordination sphere, with its backbone carbonyl oxygen acting as a ligand. Except for the second simulation, where N127 provides some additional stabilization to the metal ion, compared to the C132A site, the metal site is stable with at least three protein ligands (four in the first trajectory, where the carbonyl oxygen of G147 also enters the coordination sphere). The results are consistent with the previous electrophysiological findings.

#### 2.2.4. Solvation Analysis

**Figure 5:**
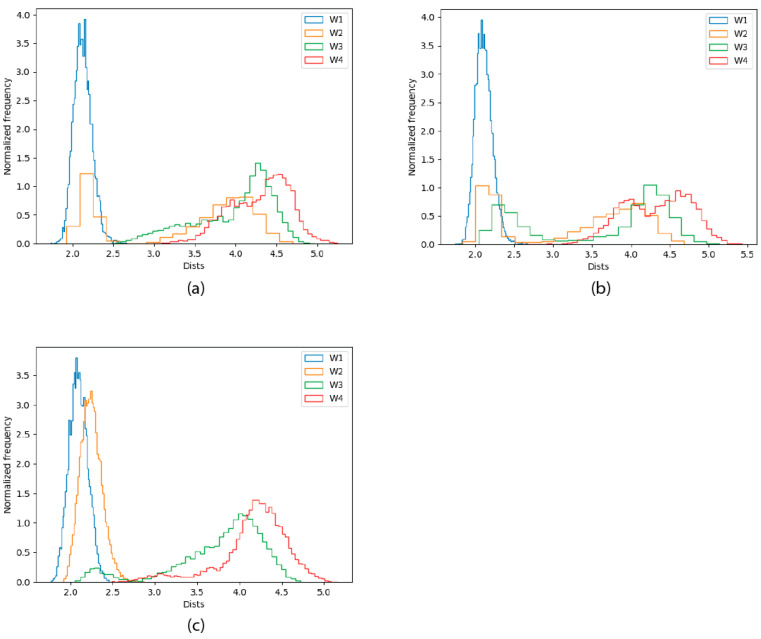
Frequency histograms for the distances of the four closest water molecules to the Zn(II) ion (named starting from the closest, W1 through W4), and the metal in the 30 ps QM/MM MD simulations. (**a**) WT trajectories; (**b**) C132A trajectories; (**c**) C132A/C159A trajectories.

Our results indicate a reduction in the number of protein ligands in the coordination sphere of the C132A mutant, which is not observed in the WT P2X4R, and is observed to a lesser extent in the double mutant C132A/C159A variant. Although Figure 4, and the equivalent figures for the other C132A trajectories (Figure S1), clearly show a displacement of Zn(II) towards the solvent bulk along the simulation, we measured the distance of the four closest water molecules to the metal ion in each point of each trajectory, and aggregated the three trajectories for each variant and the frequencies observed for each distance, in a histogram for each variant (Figure 5).

The figure shows that the metal site contains two water molecules in the WT trajectories, with one of them being much more stable in the site than the other. For the C132A mutant, three water molecules enter the coordination site, with the first one being the most stable. The situation goes back to a WT-like state for the C132A/C159 mutant, where the occupancy of the third water molecule is reduced (the remaining occupation is related to the behavior of the second trajectory for that variant, as shown in Figure 4). Altogether these data strongly suggest that the presence of C132 is critical for the coordination of the metal, and in its absence the metal starts escaping to the solvent. When both C132 and C159 thiols are mutated, new residues enter the coordination sphere and Zn(II)’s coordination sphere remains primarily composed of amino acid residues (as opposed to water molecules).

#### 2.2.5. Global Dynamical Features

**Figure 6:**
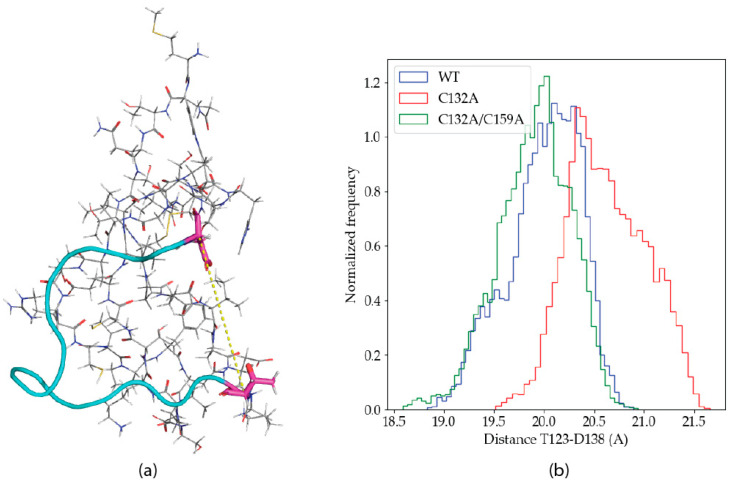
(**a**) Angular conformation of the 122–151 subsequence, in cyan, and the T123 (down) and D138 residues, in magenta. The distance between the respective alpha carbons is shown as a yellow dashed line. (**b**) Histogram of the T123-D138 distances for the aggregated wild type, C132A and C132A/C159A trajectories.

The experimental solution structure for the P2X4R head domain [21] (PDB ID: 2RUP) reveals an angular conformation in the subsequence 122-151. Visual inspection of the trajectories suggests that the angle is modified by Zn(II) binding. In order to study a possible conformational change upon metal binding, the distance between the alpha carbons of the residues T123 and D138 were measured along the trajectories, as a proxy of the mentioned angle. Figure 6a shows the angular structure in 122–151, as well as the location of T123 and D138.

Figure 6b shows the frequency distribution for the distance between the T123-D138 alpha carbons in the aggregated trajectories of the different P2X4R variants studied. The histograms show a clear difference in the distribution of the distances, where C132A shows an increased frequency for higher values. The difference in the distances observed is small, but consistent. It is unlikely that a large conformational transition would be observed in the limited simulation times attainable with the QM/MM methodology, and the computational power available today.

## 3. Discussion

The Zn(II) binding site on P2X4R has remained elusive for years, although several pieces of evidence indicate that Cys132 plays a key role in metal coordination including electrophysiological and in silico data [22,23,24]. In accordance with crystallographic structure of the *Danio rerio* P2X4R [25,26] plus the crystallographic structures of the *Amblyomma maculatum* P2X, very similar to P2X4R [27] and the P2X3R [28] proteins, the conserved head domain Cys forms five disulphide bonds, as is normally expected for extracellular cysteines. Interestingly, in the open and closed structure of the full length P2X7R [29], the Cys from SS2 and SS3 are in a reduced and oxidized form, respectively. In this context, it was proposed [20] that some of these cysteines are not physiologically oxidized, with a fraction of them in their reduced state. The hypothesis is supported by their susceptibility to alkylation by reagents such as N-ethylmaleimide or iodoacetamide in the presence of 10 micromolar extracellular Zn(II). A few examples exist of extracellular proteins where a fraction of the cysteines is in the reduced form (not forming a disulfide bond) [30]. In this work, we addressed this problem by using the GFN2-Xtb [31] semiempirical method, taking advantage of the largely systematic nature of its errors in the description of chemical reactions. Indeed, we observed that the free energy for the reduction of C126, C132, C149 and C159 is −22 kcal/mol, a value consistent with a reduced form of the protein present in the equilibrium, and with our previous data.

The present results show, for the first time, an octahedral Zn(II) coordination sphere in the P2X4R head domain encompassing C126, N127, C132, C149, C159 plus a water molecule, in agreement with electrophysiological experimental data [20]. C132 has been shown to be critical for Zn(II) positive modulation [22,24,25]. Our simulations show that the Zn(II) coordination site is not stable in the C132A mutant, in agreement with previous computational results [24]. Our data indicates that two out of the four remaining ligands abandon the coordination sphere in this mutant, displacing the metal towards the solvent. Thus, the C132 thiol is critical to maintain the Zn(II) coordination sphere integrity. Consistent with this proposal, we observed an increase in water molecules surrounding the metal, within less than 3 Å, in the C132A mutant. An effect not observed in the WT P2X4R.

Another feature of this work refers to the plausible explanations for the finding that the P2X4R is Zn(II)-modulated even when Cys132 and Cys159 are mutated, but not when C132 alone is mutated. Electrophysiological findings showed that the double mutants C132A/C159A, C132T/C159T and C132S/C159S are positively allosterically modulated by the metal. The present simulations clearly show that in the C132A/C159A double mutant, R148 and G147 become part of the coordination sphere when both Cys132 and Cys159 are simultaneously mutated to alanine. Thus, the data supports the idea that the C132 and C159 thiols may be replaced in the coordination sphere by neighboring residues when both thiols are absent. We also observed that the T123-D138 region forms an angle that closes when Zn(II) is in the coordination sphere, both in the wild type and C132A/C159A double mutant, but to a lesser extent in the single C132A mutant. Although the statistical significance of the observed transition is difficult to assess and cannot be considered quantitative due to the short simulation times allowed by the methodology, it provides a plausible hypothesis for the structural effect of Zn(II) binding to the protein, which would originate the allosteric effect observed experimentally.

While our simulated times are orders of magnitude shorter than modern classical MD standards, they are in line with what is attainable with the much more computationally demanding QM/MM methodology employed, particularly given the size of the quantum-chemical systems. Despite their limitations, the present calculations allowed visualization of a labile octahedral Zn(II) coordination sphere that includes four cysteines from the receptor head domain, in agreement with the reversibility of the metal modulation observed. The observed changes, except for the conformational displacement reported in Figure 6, are all fast transitions, expected to be sampled in the time-scale employed, as our results indeed suggest.

## 4. Materials and Methods

All models for the head receptor domain of the P2X4R were built from the solution structure for the domain of the *Rattus norvegicus* protein [21] (PDB code: 2RUP). The models included the full 58 aminoacidic residues of the receptor head.

### 4.1. Estimation of Redox Potentials

To estimate the overall redox potential of the cysteine residues of the putative Zn(II) binding domain of P2X4R (C126, C132, C149 and C159), two models were constructed. One was a fully oxidized model, the state observed in the experimental structure [21], where the C126-C149 and C132-C159 pairs form disulfide bridges. The other was a fully reduced model, where the aforementioned cysteines are in their thiol form. The C116 and C165 residues, which are away from the putative Zn(II) site, were left in their experimental forms, forming a disulfide bridge, in both models.

The obtained oxidized and reduced models were fully optimized with the semiempirical GFN2-xTB method [31], employing the ALPB continuum solvent model for water, as implemented in the xtb program [32,33]. Frequencies were obtained at the same level of theory for the full models, and free energies were estimated from the electronic, solvation and vibrational contributions. Thus, the Gibbs free energy for the half reaction of the putative zinc site reduction was obtained.

While the GFN2-xTB method is not parameterized for electronic energies, and does not produce accurate bond-breaking energies, the errors of the GFN methods are known to be, to a large extent, systematic [31,34]. To mitigate the systematic part of these errors, the other half reaction was chosen as the oxidation of glutathione, where a disulfide bridge is also formed. Thus, the full reaction consists of of the oxidation of four glutathione molecules to reduce the four cysteine residues of the putative Zn(II) site. The free energy for the glutathione oxidation half reaction was estimated with the same methodology described. The oxidized and reduced glutathione molecules were optimized and had their frequencies calculated with GFN2-xTB and the ALPB solvent model for water.

### 4.2. Structural and Dynamical Characterization of the Putative Zn(II) Site of P2X4R in Its WT, and Mutant Variants

Models were constructed for the C132A and C132A/C159A mutants of P2X4R by starting with a fully reduced putative Zn(II) site and manually replacing the thiol group in the target cysteine residues with a hydrogen. A Zn(II) ion was placed near its putative binding site.

QM/MM models were constructed for the three structures (WT and mutants) by employing GROMACS [35] software to build a solvation box of TIP3P [36] waters for each model, plus Na^+^ and Cl^−^ ions to a concentration of 0.1 M on top of those required for neutralization. The AMBER99SB-ILDN [37] force field for the protein itself. The box’s geometry was optimized classically with Gromacs. After the preliminary optimization, the system was read into the pDynamo [38] QM/MM engine by employing the ParmEd [39] tool. All QM/MM MD calculations were subsequently performed with pDynamo. A sphere was cut by selecting all residues with at least an atom within 29 Å of the alpha-carbon of GLU120, considered to be reasonably close to the centroid of the protein. The sphere was chosen in such a way that the whole protein was hydrated. The system was subdivided into a QM and a MM part. The QM subsystem was defined as the subsequences 124–134, 147–151 and 157–161, in addition to the Zn(II) ion. Any water with at least an atom within 3 Å of any atom in the aforementioned QM subsystem was also added to it, which was shown previously to be a reasonable cutoff [40]. Thus, the final QM systems comprised 563 atoms for the WT structure, 517 for the C132A mutant and 522 for the C132A/C159A mutant structure. The QM systems were chosen to be neutral and to contain all cysteine residues under study (and their corresponding alanine residues in the mutants) plus at least two residues in the sequence between each cysteine and the MM system. Spherical boundary conditions were employed in the subsequent calculations, with every atom assigned a potential of 0 inside a sphere of radius of 30 A, centered on the initial position of the alpha carbon of GLU120, and 10 kJ/mol outside the sphere.

The three models were treated with the additive QM/MM scheme implemented in pDynamo, employing the aforementioned force-fields and the semiempirical GFN2-xTB method for the MM and QM subsystems, respectively. The PQMT script (http://github.com/rmera/pqmt, last accessed on 20 June 2021) was used to connect the xtb and pDynamo programs. Three sets of geometry optimizations, plus equilibration molecular dynamics of between 1 and 10 ps were run for each structure to a final temperature of 310 K. A NVT Langevin MD simulation of 30 ps was performed on each equilibrated structure, yielding a total of nine QM/MM-MD trajectories (three for each protein variant in study). The goMD program (http://www.github.com/rmera/gomd, last accessed on 20 June 2021), which uses the goChem library [41] was employed for the analysis of the simulation data. PyMOL [42] and Matplotlib [43] were employed to prepare the figures. All programs employed, including our pqmt script, are open-source, and freely available at their respective webpages.

## 5. Conclusions

We present a computational biochemistry study of the Zn(II) binding to the PX4R head domain, at the quantum and hybrid QM/MM levels. Our approach provides data confirming previous experimental findings that used thiol chemical reagents to tentatively identify putative Zn(II) binding sites involved in the positive allosteric modulation of P2X4Rs. The combination of electrophysiology plus computational strategies fostered a dynamic approach of events occurring in the psec scale supporting few Amstrong’s transitions in the 3D protein structure compatible with the physicochemical principles that operate life machinery and consistent with biological reactions.

## Data Availability

All data from this study are available upon request.

## Supplementary Materials

The following are available online at https://www.mdpi.com/article/10.3390/ijms22147288/s1.
