# Supplementary material for: Hybrid QM/MM Simulations Confirm Zn(II) Coordination Sphere That Includes Four Cysteines from the P2 × 4R Head Domain
Source: Int J Mol Sci. 2021 Jul 7;22(14):7288. doi: 10.3390/ijms22147288 (PMC8303255; doi:10.3390/ijms22147288)
Supplement: Supplementary file 1 [file ijms-22-07288-s001.zip › ijms-1254522-supplementary.pdf]

International Journal of
Molecular Sciences

MDPI

Supplementary information for:

Hybrid QM/MM simulations confirm Zn(II) coordination sphere that includes four cysteines from P2X4R head dom

Francisco Andrés Peralta, Juan P. Huidobro-Toro and Raúl Mera-Adasme

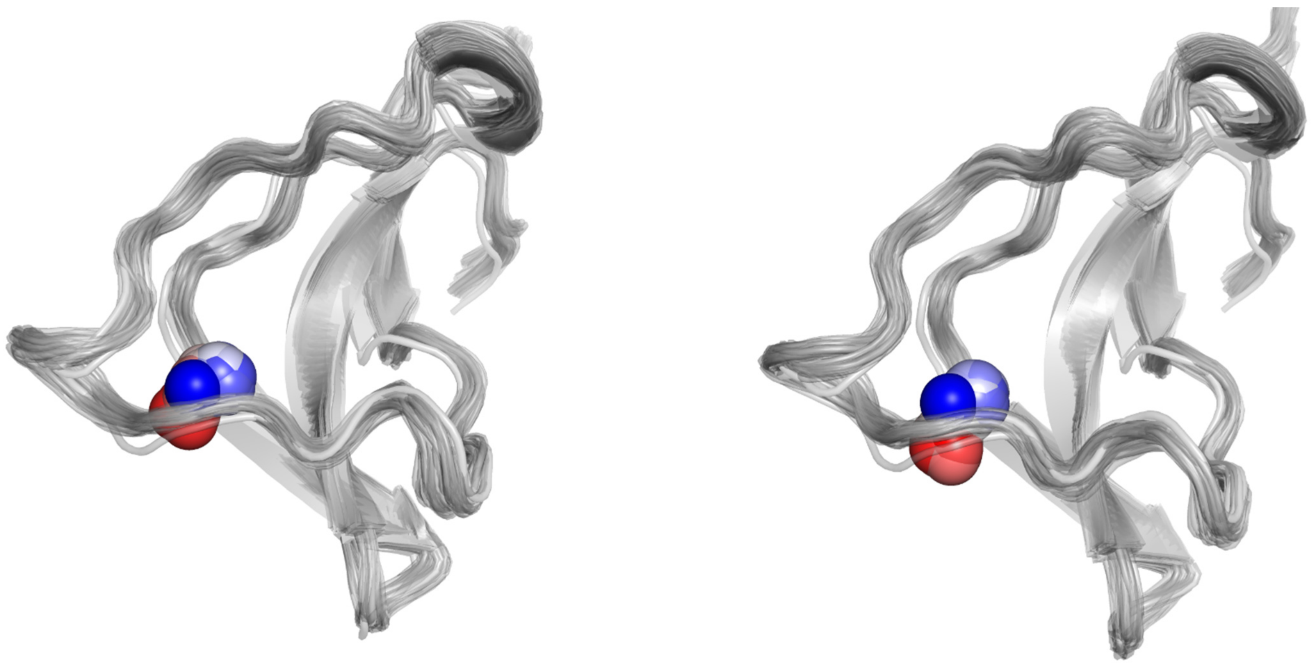

Figure S1: Displacement of the Zn(II) ion towards the solvent bulk. Displacement of Zn(II) along the C132A QM/MM MD trajectories not shown in the main document. The different positions of the Zn(II) ion are shown with spheres colored from blue (for the position at the beginning of the simulation) to white, to red (at the end of the simulation).
